# The prevalence and risk factors of burnout and its association with mental issues and quality of life among hungarian postal workers: a cross-sectional study

**DOI:** 10.1186/s12889-023-15002-5

**Published:** 2023-01-11

**Authors:** Miklós Kovács, György Muity, Ádám Szapáry, Zsolt Nemeskéri, Imre Váradi, Krisztián Kapus, Antal Tibold, Nikoletta Magyar Zalayné, Lilla Horvath, Gergely Fehér

**Affiliations:** 1 Centre for Occupational Medicine, Medical School, University of Pécs, 7624 Pecs, Hungary; 2 Harkany Spa and Thermal Center, Harkány, 7815 Hungary; 3 Faculty of Cultural Sciences, Education and Regional Development, University of Pécs, 7633 Pecs, Hungary; 4 Department of Primary Health Care, University of Pécs, 7623 Pecs, Hungary

**Keywords:** Burnout, Depression, Insomnia, Risk factor, Prevalence, Quality of life, Cross sectional study

## Abstract

**Background:**

Burnout is one of the most extensively studied phenomena of the twenty-first century; which has been extensively studied among helping professions, although it can be broadened to several other types of occupation. Based on our knowledge and literature search, no similar studies have been carried out among postal workes to date.

**Methods:**

This cross-sectional questionnaire-based epidemiological study was carried out between May 2021 and January 2022 in five counties in Hungary with the recruitment of postal delivery workers focusing on (1) the prevalence of burnout among postal delivery workers; (2) including the role of demographic parameters, duration of employment as well as the presence of secondary employment; (3) and also analyzed the role of several risk factors and medical conditions; (4) and we also examined the possible association between depression, insomnia and quality of life and burnout.

**Results:**

Overall 1300 questionnaires were successfully delivered and 1034 responses received (response rate of 79.5%). Three hundred sixty-eight males (35.6%) and six hundred sixty-six females (64.4%) participated in our study. The prevalence of burnout was 50.8% (525/1034) in this study population (mean score 2.74 ± 0.33). Logistic regression analysis showed that female gender [OR = 2.380, 95% CI: 1.731 to 2.554], first workplace [OR = 1.891, 95% CI: 1.582 to 2.162] and working more than 30 years [OR = 1.901, 95% CI: 1.608 to 2.326] have significantly increased the likelyhood of burnout as well as the history of muscoskeletal pain [OR = 1.156, 95% CI: 1.009 to1.342], current quality of life [OR = 1.602, 95% CI: 1.473 to 1.669] and the presence of sleep disturbance [OR = 1.289, 95% CI: 1.066 to 1.716].

**Conclusion:**

This is the first study in Hungary to investigate the prevalence of burnout among postal workers and to explore the relationship between burnout and mental health problems. Our study underlines the clinical importance of burnout and draws attention to the need for appropriate prevention and treatment strategies.

## Introduction

Burnout is one of the most extensively studied phenomena of the twenty-first century; it most commonly affects individuals whose work requires interpersonal contact and empathy (such as healthcare workers and teachers), but later evidence has shown that this phenomenon can develop (although relatively poorly studied) among those who were not initially considered at risk such as manual workers [1, 2]. Cumulative evidence has shown that burnout is an emotional, mental and physical exhaustion provoked by prolonged emotional strain and stress, associated with behavioural and self-esteem disorders, impaired coping strategies [3].

Work-related conditions are the most important in the development of burnout; overwork, increased workplace stress, lack of effort and rewards and imparied collegial relationships are well-known precursors [2–5]. Relatively recent studies have also showed the important role of certain individual traits are not negligible precedessors such as emotional labor, maladaptive traits or neuroticism [3, 4, 6, 7]. Sociodemographic factors such as younger age, being single and having less work-related experience can also be important predictors [7, 8].

Burnout can have serious consequences, it can have enormous effects both on the individual and the society due to personal and interpersonal disturbances [8–10]. It can also lead to serious health issues, such as insomnia, increased anxiety and depression and can also be closely associated with cardiovascular complications, chronic pain syndromes and increased sudden mortality in the young (< 45 years) [11–14]. Considering the adverse effects of burnout as seen above, it can negatively impact all domains of life and can be associated with lower quality of life, although this connection has rarely been studied among “blue-collar” workers [2, 15–18].

The absence of an objective measurement tool also complicates the study of this phenomenon. The most widely used questionnaire is the Maslach's Burnout Inventory (MBI), which was considered to be the’’gold-standard” of measurement. However, the psychometric properties of the Copenhagen Burnout Inventory (CBI) or the Oldenburg Burnout Inventory (OLBI) seemed to be more reliable in the detection of burnout based on a recent analytic study [19].

Burnout is extensively studied among helping services, although it can be broadened to any occupations including blue collar workers. Based on our knowledge and literature search, no similar studies have been carried out among postal workes. The Hungarian Post is the largest state-owned company in our country, with approximately 40,000 employees. Based on their official website, they have to face an increased rate of workplace fluctuations, according to their report, the turnover rate was 23.5% in 2018, which has led to the launching of various staff retaining programmes with very modest results [20]. The main reported reasons of leaving were low salaries and work overload, which are also potential risk factors for burnout.

Based on the above, we carried out a questionnaire based survey which was cross-sectional in nature, focusing on (1) the prevalence of burnout among postal delivery workers; (2) including the role of demographic parameters such gender, age, marital status, number of children, duration of employment as well as the presence of secondary employment; (3) and also analyzed the role of risk factors and medical conditions such as smoking, alcohol and drug consumption, diabetes, hypertension, cardiovascular disease, musculoskeletal pain and history of depression. We also examined the possible association between depression, insomnia and quality of life and burnout.

## Methods

This cross-sectional questionnaire-based epidemiological study was carried out between May 2021 and January 2022 in five counties in Hungary recruiting postal delivery workers. The study was approved by the Ethics Committee of the University of Pécs and confirmed by the general and local management of the Hungarian Post Office (reference number: PTE/96773–2/2018). Participation was anonymous and voluntary.

Inclusion criteria were having a signed consent before entering the study, being employed (as public servant, subcontractor, etc.) as a postal delivery worker, age between 18 and 65 years and being at work at the time of the study. Exclusion criteria were the absence of a signed consent, refusing to participate in the study, not being a delivery worker, being on permanent leave or being younger than 18 or older than 65 years of age.

Demographic data included gender, age, marital status, number of children, duration of employment and number of workplaces as well as the presence of secondary employment (taking on a second job). Risk factors included were everyday tobacco use, regular alcohol intake (regular and/or having drinking, overall taking more than 20 units pro week), and any time of illicit drug use. Medical conditions taken into account were the presence of diabetes, hypertension, ischemic heart disease, history of musculoskeletal pain and depression.

Burnout was measured by the Mini Oldenburg Burnout Inventory (MOLBI), which has shown to have robust psychometric properties in the measurement of occupational burnout (Cronbach’s alpha 0.806) [21, 22]. The advantage of this questionnaire includes that it can be used as a universal measurement tool for any profession and it was specifically designed to reduce the content and theoretical criticisms of the MBI questionnaire. Thus, the statements are specific and their number is evenly distributed within each scale, with an even distribution of positive–negative items. The questionnaire measures burnout along two dimensions: exhaustion measures work-related fatigue and the emotional, cognitive and physical strain of work; the disillusionment scale measures loss of interest in work, depersonalisation of work, loss of commitment and possible cynicism. Half of the them are reversed and answers are scored on a four-point Likert-type scale, and high scores for the degree of burnout here also indicate a high degree in the two subscales [21]. Mean scores ≥ 2.25 on exhaustion and ≥ 2.1 on disappoinment considered to be high and job burnout was diagnosed to be fulfilling both criteria [21, 22]. This questionnaire is validated in Hungarian language [22].

The presence and severity of depression were screened by the short version of the Beck Depression Inventory (BDI-SF) (Cronbach’s alpha 0.920) [23, 24]. This questionnaire contains 9 items on the following symptoms: social withdrawal, indecision, sleep disturbance, fatigue, excessive worry about physical symptoms, inability to work, pessimism, dissatisfaction, lack of pleasure. The scale is scored from 1 to 4 points, and we can distinguish between the absence (0–9 points) and the presence of depression (> 10 points) and depression can be cathegorized into mild (10–18 points), moderately severe (19–25 points) and severe (≥ 26 points) forms. This questionnaire was validated for use in Hungary [23].

Sleep disturbance was measured using the Athens Insomnia Scale (AIS) (Cronbach’s alpha 0.878), this questionnaire comprises eight questions, five of which measure nocturnal symptoms (difficulty falling asleep and staying asleep, early awakening) and three ask about daytime consequences [25]. The higher the score, the worse the sleep quality (up to a maximum of 24 points). A score of 10 indicates clinically significant insomnia [25]. This questionnaire is also available in Hungarian language [26].

Quality of life was surveyed using the EQ-5D (health-related quality of life) self-completion questionnaire, which is widely available in several languages (Cronbach’s alpha 0.856). The questionnaire measures 5 dimensions (mobility, self-care, usual activities of daily living, pain/discomfort, and anxiety/depression), with a set of statements corresponding to a 3-point scale [27].

Statistical analysis: Study population data were analyzed as means ± SD (standard deviation) using chi-square test, distribution ratios. Data were evaluated with the use of descriptive statistics, analysis of variance, correlation ratios and logistic regression analysis. The potential differences between males and females were analyzed with the use of T-test and Chi‐square test. The analysis of variance was used to evaluate the differences in the study subgroups (absence of burnout, emotional exhaustion, disappointment and job burnout) including sociodemographic factors, prevalence of mental health problems, sleep quality, comorbidity. The association burnout and depression, sleep disturbance and quality of life score was explored using Pearson’s correlation coefficients. A logistic regression analysis was also employed to identify the impact of predictor variables. A confidence interval (CI) of 95% was set up in our study for all odds ratios. Statistical analysis including the above mentioned methods and analysis were carried out with the use of the statistical package of SPSS 11.0 (SPSS, Chicago, IL, USA).

## Results

Overall, 1300 questionnaires were successfully delivered and 1034 responses received (response rate of 79.5%). Three hundred and sixtyeight males (35.6%) and six hundred sixty-six females (64.4%) participated in our study. Vast majority of our study participants were between 26–45 years of age (67.4%). Seven hundred sixty-four people (73.89%) were married or lived in a relationship. The number of childless workers was 236 (22.8%). The vast majority (45.8%) of our study population had been working for 21–40 years. Fifty-eight participants (5.6%) had another part-time job (Table 1). Four hundred forty-five (43.0%) were on regular medication. The number of regular smokers was 299 (28.9%) and 91 (8.8%) were using alcohol regularly. Only 34 (3.3%) have ever tried the use of drugs. Three hundred and five (29.5%) participants had hypertension, 185 (17.9%) had musculosceletal pain. Ischemic heart disease was be detected in 124 (12.0%) workers. 62 (6.0%) participants had diabetes (Table 2).

**Table 1:** Distribution of sociodemographic parameters and its association with burnout in males and females

(Data %. *N* = 1034)	**Study population** **(1034)**	**Male** **(368/1034)**	**Female** **(666/1034)**
**Age**			
18–25 years	4.0 (41/1034)	3.5 (13/368)	4.2 (28/666)
26–35 years	13.3 (138/1034)	14.7 (54/368)	12.6 (84/666)
36–45 years	23.5 (243/1034)	19.8 (73/368)	25.5 (170/666) ^#^
46–55 years	43.9 (454/1034)	41.0 (151/368)	45.5 (303/666)
56–62 years	13.7 (142/1034)	17.7 (65/368)	11.6 (77/666) ^#^
more than 62 years	1.6 (16/1034)	3.3 (12/368)	0.6 (4/666) ^##^
**Marital status**			
single	12.9 (133/1034)	18.8 (69/368)	9.6 (64/666) ^##^
relationship	17.1 (177/1034)	17.2 (63/368)	17.1 (114/666)
married	56.8 (587/1034)	54.7 (201/368)	58.0 (386/666)
divorced / widow	13.2 (136/1034)	9.3 (34/368)	15.3 (102/666) ^#^
**Number of children**			
have no child	22.8 (236/1034)	29.6 (109/368)	19.1 (127/666) ^##^
1 child	26.0 (269/1034)	23.9 (88/368)	27.2 (181/666)
2 children	37.2 (385/1034)	31.8 (117/368)	40.2 (268/666) ^#^
> 3 children	13.9 (144/1034)	14.7 (54/368)	13.5 (90/666)
**Workplace**			
1st	40.0 (414/1034)	24.7 (91/368)	48.5 (323/666) ^##^
2nd	19.0 (196/1034)	23.6 (87/368)	16.4 (109/666) ^#^
3rd	17.7 (183/1034)	18.5 (68/368)	17.3 (115/666)
4th	9.1 (94/1034)	11.7 (43/368)	7.6 (51/666) ^#^
5th or more	14.2 (147/1034)	21.5 (79/368)	10.2 (68/666) ^##^
**Years spent with work**			
1–12 months	4.5 (45/1034)	4.9 (18/368)	4.1 (27/666)
1–5 years	20.3 (210/1034)	19.0 (70/368)	21.0 (140/666)
6–10 years	10.3 (107/1034)	12.2 (45/368)	9.3 (62/666)
11–20 years	17.5 (181/1034)	17.4 (64/368)	17.5 (117/666)
21–30 years	25.6 (265/1034)	26.1 (96/368)	25.4 (169/666)
31–40 years	19.9 (206/1034)	17.7 (65/368)	21.2 (141/666)
more than 40 years	1.9 (20/1034)	2.7 (10/368)	1.5 (10/666)
**Secondary employment**			
No	94.4 (976/1034)	91.0 (335/368)	96.2 (641/666)
Yes	5.6 (58/1034)	9.0 (33/368)	3.8 (25/666) ^#^

^#^*p* < 0.05 between males vs females

^##^*p* < 0.001 between males vs females

**Table 2:** Distribution of risk factors and medical conditions and their association with burnout in males and females

(Data %. *N* = 1034)	**Study population (1034)**	**Males** **(368/1034)**	**Females** **(666/1034)**
**Comorbidities**			
on regular medication	43.0 (445/1034)	44.3 (163/368)	42.3 (282/666)
smoker	28.9 (299/1034)	34.0 (125/368)	26.1 (174/666) ^#^
alcohol use	8.8 (91/1034)	21.5 (79/368)	1.8 (12/666) ^##^
drug use	3.3 (34/1034)	5.4 (20/368)	2.1 (14/666) ^#^
diabetes	6.0 (62/1034)	7.6 (28/368)	5.1 (34/666)
hypertension	29.5 (305/1034)	36.4 (134/368)	25.7 (171/666) ^##^
ischemic heart disease	12.0 (124/1034)	13.3 (49/368)	11.3 (75/666)
musculosceletal pain	17.9 (185/1034)	21.2 (78/368)	16.1 (107/666) ^#^
history of depresssion	4.9 (51/1034)	6.0 (22/368)	4.4 (29/666)

^#^*p* < 0.05 between males vs females

^##^*p* < 0.001 between males vs females

The prevalence of burnout was 50.8% (525/1034, 95% CI: 44.9% to 56.7%; mean score 2.74 ± 0.28, 95% CI: 2.72 to 2.77). Subgroup analysis showed that 52.8% (95% CI: 49.6% to 56.0%) suffered from exhaustion (546/1034, mean score 2.67 ± 0.306, 95%CI: 2.64 to 2.69) and 78.9% (CI 95% 71.3% to 86.5%) felt disappointed (816/1034 mean score was 2.73 ± 0.353, 95% CI: 2.71 to 2.75) (Table 3). Five hundred eighty-five (56.6%, 95% CI: 55.4 to 57.8) suffered from mild, 64 (6.2%, 95% CI: 6.07% to 6.33%) from moderate, and 10 (1%, 95% CI: 0.72% to 1.28%) from severe depression (Table 3). The prevalence of sleep disturbance was 17.9% (185/1034, 95% CI: 15.6% to 19.8% and 4.6% (48/1034, 95% CI: 3.4% to 5.6%) had severe insomnia (Table 3).

**Table 3:** The distribution of burnout, depression and insomnia in the study population and their association with gender

(*N* = 1034)	**%**	**Male** **(368/1034)**	**Female** **(666/1034)**
**Burnout**			
burnout	50.8 (525/1034)	47.5 (175/368)	56.3 (350/666) ^#^
exhaustion	52.8 (546/1034	49.5 (182/368)	54.7 (364/666)
disappointment	78.9 (816/1034)	74.5 (274/368)	81.4 (542/666) ^#^
**Depression**			
normal	36.2 (375/1034)	40.5 (149/368)	33.9 (226/666) ^#^
mild	56.6 (585/1034)	51.6 (190/368)	59.3 (395/666) ^#^
moderate	6.2 (64/1034)	6.8 (25/368)	5.9 (39/666)
severe	1.0 (10/1034)	1.1 (4/368)	0.9 (6/666)
**Sleep disturbance**			
no	77.5 (801/1034)	81.8 (301/368)	75.1 (500/666) ^##^
present	17.9 (185/1034)	15.2 (56/368)	19.4 (129/666)
severe	4.6 (48/1034)	3.0 (11/368)	5.5 (37/666)

^#^*p* < 0.05 between males vs females

^##^*p* < 0.001 between males vs females

### Univariate analysis – males vs. females

The age group of 36–45 years was more common among females than in males (25.5% vs. 19.8%, *p* = 0.038). Being over 56 years was more common among males (17.7% vs. 11.6%, *p* = 0.06 and 3.3% vs. 0.6%, *p* < 0.001). The rate of being single was significantly increased among males (18.8% vs. 9.6%, *p* < 0.0019, while being divorced was more common among females 15.3 vs. 9.3%, *p* = 0.007). Being employed at the first worplace had female predominance (48.5% vs. 24.7%, *p* < 0.001). Secondary employment (another part time job) was significantly common among males (9% vs. 3.8%, *p* < 0.001) (Table 1). Males had significantly more common tobacco (34% vs. 26,1%, *p* = 0.008), alcohol (21.5% vs 1.8%, *p* < 0.001) and drug consumption (5.4% vs. 2.1%, *p* = 0.004) comparig to females. The rate of hypertension (36.4% vs. 25.7%, *p* < 0.001) and history of low back pain (21.2% vs. 16.1%, *p* = 0.04) was also significantly more common in males (Table 2). The rate of burnout was significantly lower among males (47.5% vs 52.6%, *p* = 0.04). The absence of depression (40.5% vs 33.9%, *p* = 0.03) and sleep disturbance (81.8% vs. 75.1%, *p* = 0.03) was significantly higher among males (Table 3).

### Univariate analysis – study population

Burnout was associated with female gender (66.7% vs 55.8%, *p* = 0.038). Being employed at the first workplace (44.6% vs 26.9%, *p* = 0.0013) as well as working for > 30 years (31–40 years and more than 40 years) was associated with burnout (19.8% vs 12.7%, *p* = 0.001, 2.9% vs 1.5%, *p* = 0.045) (Table 4). Burnout was associated with a history of musculoskeletal pain (17.3% vs. 8.6%, *p* = 0.003) and history of depression (7.6% vs. 3.5%, *p* = 0.048). Interestingly, smoking was found protective (26.1 vs 33.5%, *p* = 0.033) (Table 4). There was a significant association between burnout and severe sleep disturbance (7.1% vs 2.7%, *p* < 0.001, *r* = 0.130, *r*^2^ = 0.105, β = 0.106). Moderate depression (12.7% vs 3.3%, *p* = 0.003,, *r* = 0.083, *r*^2^ = 0.070, β = 0.083) and lower quality of life (84.74 vs. 81.07, *p* = 0.016, *r* = 0.121, *r*^2^ = 0.020, β = 0.049) were also associated with burnout (not shown).

**Table 4:** Main components of burnout in the study population

(Data %. *N* = 1034)	**No burnout** **(197/1034)**	**Exhaustion** **(546/1034)**	**Disappointment** **(816/1034)**	**Burnout** **(525/1034)**
**Gender**				
*Female*	*55.8 (110/197)*	*66.7 (364/546)*	*66.4 (542/816)*	66.7* (350/525)
Male	44.2 (87/197)	33.3 (182/546)	33.6 (274/816)	33.3 (175/525)
**Number of workplace**				
*1st*	*26.9 (53/197)*	*44.1 (241/546)*	*43.4 (354/816)*	44.6* (234/525)
2nd	25.4 (50/197)	16.1 (88/546)	17.4 (142/816)	16.0 (84/525)
3rd	20.8 (41/197)	16.5 (90/546)	17.2 (140/816)	16.8 (88/525)
4th	11.2 (22/197)	8.8 (48/546)	8.5 (69/816)	8.6 (45/525)
5th or more than 5th	15.7 (31/197)	14.5 (79/546)	13.6 (111/816)	14.1 (74/525)
**Years spent with work**				
1–12 months	5.1 (10/197)	4.8 (26/546)	4.3 (35/816)	5.0 (26/525)
1–5 years	28.4 (56/197)	17.8 (97/546)	18.4 (150/816)	17.7 (93/525)
6–10 years	12.2 (24/197)	9.5 (52/546)	9.9 (145/816)	9.5 (50/525)
11–20 years	17.3 (34/197)	18.1 (99/546)	17.8 (213/816)	18.5 (97/525)
21–30 years	22.8 (45/197)	26.9 (147/546)	26.1 (175/816)	26.7 (140/525)
*31–40 years*	*12.7 (25/197)*	*20.1 (110/546)*	*21.4 (17/816)*	19.8* (104/525)
*more than 40 years*	*1.5 (3/197)*	*2.7 (15/546)*	*2.1 (17/816)*	2.9* (15/525)
**Comorbidities**				
taking medication regularly	45.2 (89/197)	43.0 (235/546)	42.2 (344/816)	42.5 (223/525)
*cigarette smoker*	*33.5 (66/197)*	*26.7 (146/546)*	*27.5 (224/816)*	26.1* (137/525)
taking any alcohol	10.7 (21/197)	9.0 (49/546)	8.1 (66/816)	8.6 (45/525)
taking any drugs	4.1 (8/197)	2.7 (15/546)	3.2 (26/816)	2.9 (15/525)
diabetes	7.6 (15/197)	6.2 (34/546)	5.8 (47/816)	6.5 (34/525)
hypertension	30.5 (60/197)	28.6 (156/546)	28.9 (236/816)	28.0 (147/525)
ischemic heart disease	10.2 (20/197)	11.4 (62/546)	12.4 (101/816)	11.2 (59/525)
history of musculosceletal pain	17.3 (34/197)	14.7 (80/546)	18.1 (148/816)	14.7 (77/525)
history of depression	7.6 (15/197)	4.2 (23/546)	4.2 (34/816)	4.0 (21/525)

^*^p < 0.05)

### Multivariate analysis

A logistic regression performed to ascertain the effects of gender, number of workplace, time spent with work, history of musculoskeletal pain, history of depression, smoking, sleep disturbance, depression and quality of life on the likelyhood of the presence of burnout versus the absence of this phenomenon. The logistic regression model was statistically significant (*p* = 0.000, *X*^2^ = 31.85). The model explained 5.8% (Nagelkerke R^2^) of variance in burnout and correctly classified 72.0% of cases. Female gender [OR = 2.380, 95% CI: 1.731 to 2.554], first workplace [OR = 1.891, 95% CI: 1.582 to 2.162] and working more than 30 years [OR = 1.901, 95% CI: 1.608 to 2.326] have significantly increased the likelyhood of burnout as well as the history of muscosceletal pain [OR = 1.156, 95% CI: 1.009 to1.342], current quality of life [OR = 1.602, 95% CI: 1.473 to 1.669] and the presence of sleep disturbance [OR = 1.289, 95% CI: 1.066 to 1.716] (Table 5).

**Table 5:** Predictors of burnout in a logistic regression analysis

*N* = 1034	B	SE B	p	OR	CI 95% Lower	CI 95% Upper
Gender*	0.148	0.178	0.037	2.38	1.731	2.554
First workplace*	0.021	0.130	0.03	1.891	1.582	2.162
*Working* > 30 years*	0.062	0.060	0.006	1.901	1.608	2.326
History of musculosceletal pain*	0.762	0.445	0.038	1.156	1.009	1.342
History of depression	0.765	0.341	0.072	-	-	-
Smoking	0.310	0.115	0.096	-	-	-
Sleep disturbance*	0.083	0.028	0.02	1.289	1.066	1.716
Depression	0.006	0.005	0.205	-	-	-
Quality of life*	0.017	0.025	0.002	1.602	1.473	1.669

*p<0.05

## Discussion

This is the first study to our knowledge, which focuses on burnout among postal workers with a complex methodology. Nowadays, burnout is an increasingly widespread phenomenon, predominantly affecting those working in the human services sector, whose daily work requires more empathy and patience [28, 29]. Despite its increasing prevalence and its negative impact on health (depression, sleep disturbance, anxiety, etc.), it is still considered as an occupational phenomenon. As a result, it has not been objectively measured, and we don’t have clear interventions and guidelines for its adequate management. Burnout can be associated with somatic and psychological complications and can also have a negative impact on the social life of the individual, consequently, the main challenge lies in its prevention [30, 31].

In our study, the prevalence of overall burnout was 50.8%, which is a surpsisingly high rate, much higher than among healtcare professionals who are amongst the most vulnerable ones [3, 15, 31–33]. Burnout can have serious mental and physical consequences including mental issues as insomnia or mood disorders and can also be closely associated with cardiovascular complications, chronic pain syndromes and increased sudden mortality in the young (< 45 years), which underlines the importance of proper screening [11–14]. However, this rate is comparable to the results of our previous Hungarian studies focusing on different population of workers [26, 34, 35]. Similar to healthcare workers, their salaries are far below the Hungarian average and they also tend to being overwhelmed with work, which are the most likely explanations of higher burnout rates in these populations.

Female gender was an independent predictor of burnout in our current study. The explanation for this could be that women in addition to work are also responsible for a major part of housework and raising children, so they become emotionally exhausted sooner [2, 34]. Based on the results provided by Eurostat, there is a gender gap in time spent with household and family care activities in favor of women in all member states of the European Union [36].

Working in the first job was also a significant predictor of burnout, which is not surprising. Starting a career and thus apparently being inexperienced, lacking routine, having to cope with increased stress levels and their connection with burnout have already been described before [2, 34, 37].

There are conflicting results in the literaure with regards to the effect of years spent with work on the occurrence of burnout [38, 39]. In our study population, a significant increase of burnout rate was found among those working for > 30 years, which remained a significant predictor of the phenomenon. Considering the lack of correlation with the number of previous workplaces and the fact that having the first workplace also proved to be a predictive factor in the development of the burnoutdraws attention to the importance of proper workplace prevention.

In our study smoking was found to be a protective factor for burnout. This is slightly controversial compared to previous results as it had been shown to have no or negative effect on the prevalence of the syndrome [36, 37]. A recent study showed that emotional exhaustion, which is possibly the main component of burnout can be associated with increased rate of tobacco use, so it can be a form of coping with stress and people who are on the verge of burnout may resort to the habit quite often ([40–42], https://studenttheses.universiteitleiden.nl/access/item%3A2661447/view). We speculate that the higher rate of smoking can be attributable to increased stress, but these workers have not yet reached total burnout but were on the way to this state. Interstingly, only small, but significant association was found between in the case of alcohol consumption and burnout in a recent meta-analysis and the found effect could be considered as small based on their results ([40], https://studenttheses.universiteitleiden.nl/access/item%3A2661447/view). So the association of burnout and tobacco comsumption (and alcohol comsumption) merits further investigation. However, smoking was not a significant contributor to burnout in a multivariate analysis.

Musculoskeletal pain was significantly associated with burnout in both uni- and multivariate analysis. Faulty postures are commonly recognized among delivery workers due to the need of static work postures and lack of dynamic strength required for fine motor movements. Furthermore, increased stress and related physical and mental exhaustion can lead to increased production of cortisol, which counteracts on muscle tissues causing increased muscle tone as well as reduced strength and functioning [43, 44].

History of depression was significantly common among those who suffered from burnout and our study showed an increased prevalence of current moderate and major depression in this population, furthermore, the severity of depression significantly correlated with the severity of burnout. Depression is a condition that limits individuals in all aspects of life and is a major social burden. It is estimated to become the leading cause of disability and one of the leading causes of death by 2030 because of increased suicide rates [45]. It is a matter of debate whether there is a significant ovelap between burnout and depression [14]. On the other hand, a recent meta-analysis including a moderation analysis showed different pathways, which is in concodance with our result as neither the history of depression nor the presence of a mood disorder were significantly associated with burnout in a multivariate analysis [15].

Burnout also significantly impacts quality of sleep, as chronic stress, which is the main trigger of this phenomenon, can result in insomnia [46]. Constant activation of the hypothalamic–pituitary–adrenal axis with increased rate of cortisol and other stress hormone levels can generate both burnout, depression and insomnia as mentioned above [39, 46]. Insomnia was significantly associated with burnout in both uni- and multivariate analysis so it should be a priority for postal managers to prevent or recognize occupational burnout to to provide employees with quality professional care to manage symptoms of burnout including insomnia.

Burnout can negatively effect all domains of life which was reflected in the significantly decreased quality of life. Our results indicate that it is accompanied by a marked deterioration of various spheres of life including mental and physical health, intimacy, social status, and personal safety.

Our work has certain limitations. Due to methodological shortcomings (standardised methodology) and the lack of randomised trials, the phenomenon is under intense research. Currently, there is no agreement on the classification of burnout, and the objective measurement tools to be used have not been clearly defined. Although our study involved more than 1000 postal workers, the sample is not representative and we cannot generalize our findings, and our conclusions are limited to the population included in the study. As it was a self-completion questionnaire survey, some bias is possible and we did not have detailed medical history, for example on the duration of depression and furthermore, no physical examination or follow-up were carried out. These limitations may have affected our work and findings.

## Conclusions

Overall, our study is one of the first to investigate the prevalence and risk factors of burnout and to explore the relationship between burnout and mental health conditions (depression, insomnia, quality of life) among Hungarian postal workers. Based on the results of our present study, burnout can also occur in groups not being considered at risk, at least in the same proportion as among healthcare workers who are considered as leaders of the field. Around half of the workers included in the study suffered from burnout, which highlights the importance of the issue and the need for early intervention. Our study also can help in the identification of workers being at risk, who benefit from appropriate prevention strategies and regular screening to avoid burnout, which can be associated with severe mental and physical issues.

## Data Availability

The dataset supporting the conclusions of this article is available on request to the corresponding author.
